# Research Trends in Frailty and Delirium in Older Adults: A Bibliometric Analysis

**DOI:** 10.3390/healthcare14193141

**Published:** 2026-09-22

**Authors:** Hongdi Du, Wei Liu, Yafang Zhao, Xiaoxing Lai, Chen Fu, Yan Ma, Dan Han, Jing Li, Xiaopeng Huo

**Affiliations:** 1 Department of Health Care, Peking Union Medical College Hospital, Chinese Academy of Medical Sciences & Peking Union Medical College, Beijing 100730, China; 2 Department of Ophthalmology, Peking Union Medical College Hospital, Chinese Academy of Medical Sciences & Peking Union Medical College, Beijing 100730, China; 3 Department of Neurology, Peking Union Medical College Hospital, Chinese Academy of Medical Sciences & Peking Union Medical College, Beijing 100730, China; 4 Nursing Department, Peking Union Medical College Hospital, Chinese Academy of Medical Sciences & Peking Union Medical College, Beijing 100730, China

**Keywords:** frailty, delirium, elderly, Bibliometrics, citation analysis

## Abstract

**Background/Objectives:** Frailty and delirium represent critical geriatric syndromes with profound implications for older adults’ health outcomes. This study aimed to quantitatively map global research trends and thematic developments in frailty and delirium among older adults through a bibliometric approach. **Methods:** A literature search was conducted in the Web of Science Core Collection up to 11 May 2026. Bibliometric analyses were performed using VOSviewer, CiteSpace, and the R package “bibliometrix”. **Results:** The analysis included 911 publications published between January 2006 and May 2026, involving 6805 authors from 337 journals. The USA dominated research output with 185 articles (20.3%) and the highest total citations (8507). Harvard University led institutional contributions (139 articles), while *Age and Ageing* emerged as the most influential journal (h-index = 21). Bellelli Giuseppe was the most productive and impactful author. Keyword co-occurrence analysis identified six thematic clusters: adverse outcomes and comprehensive geriatric assessment, cognitive decline and neuropsychiatric disorders, perioperative and intensive care settings, postoperative delirium and geriatric complications, prediction and validation tools, and epidemiology and management-related themes. Temporal keyword bursts revealed recent emphases on “anesthesia” (2022–2026) and “cardiac surgery” (2023–2024). **Conclusions:** This bibliometric analysis demonstrated a sustained increase and diversification in research on frailty and delirium in older adults over the past two decades. The field has evolved from foundational clinical and epidemiological studies to multidisciplinary, outcome-oriented research, with growing attention to perioperative care and comprehensive assessment.

## 1. Background

Frailty and delirium are prevalent geriatric syndromes characterized by increased vulnerability to stressors and acute cognitive disturbances, respectively [1]. Frailty manifests through symptoms such as muscle weakness, unintentional weight loss, and exhaustion, with global prevalence estimates ranging from 12% to 24% in adults aged ≥50 [2]. Delirium, marked by acute inattention, fluctuating cognition, and altered consciousness, affects 15–75% of hospitalized elderly patients, depending on clinical settings [3]. Epidemiologically, frailty and delirium share overlapping risk factors, including inflammation, vascular dysfunction, and polypharmacy. Studies showed that frailty increased the risk of delirium by two to three times [4]. The social and economic impacts are substantial: delirium in frail older adults with dementia correlates with an increase in annual healthcare costs, while prolonged frailty states extend hospital stays and elevate mortality risks.

Recent advances in frailty and delirium research highlight their interconnected pathophysiology and clinical management. Lim et al. [5] conducted a retrospective cohort study in 902 hospitalized older adults and found HFRS-measured frailty predicted delirium, longer stays, and higher costs, validating its utility in clinical risk stratification. A nationwide study of 3.5 million older adults with acute heart failure demonstrated that coexisting frailty and delirium increased in-hospital mortality odds by 12 times and non-home discharge odds by 14 times. These synergistic effects underscored the urgency of early management of geriatric syndromes in cardiology [6]. Among 148 hip fracture patients, preoperative frailty independently increased postoperative delirium risk, advocating for frailty-targeted interventions [7]. Recently, Bellelli et al. synthesized the clinical, epidemiological, and pathophysiological evidence underlying the frailty–delirium interplay, highlighting shared mechanisms including inflammation, neurodegeneration, and vascular vulnerability as key drivers of this bidirectional relationship [8]. Despite significant progress in frailty and delirium research, comprehensive analyses that integrate global research patterns are needed. A bibliometric analysis is needed to systematically delineate evolving thematic priorities, uncover underexplored domains in elderly care, and strategically direct research efforts to address the compounding risks faced by this vulnerable population.

Bibliometrics, the quantitative analysis of published literature, offers a systematic approach to mapping research trends and collaborations across scientific domains [9]. Guo et al.’s bibliometric analysis of perioperative frailty and surgery identified research hotspots and emerging trends centered on frailty risk stratification, postoperative complications, and key patient groups and syndromes such as older adults and sarcopenia [10]. Liu et al.’s bibliometric analysis on postoperative delirium and its risk factors identified research hotspots centered on elderly populations and high-risk surgical groups, with key themes including cardiac surgery, hip surgery/hip fracture, cognitive impairment, and perioperative factors such as frailty, postoperative pain, and perioperative nursing [11]. Currently, no bibliometric analysis has been dedicated to exploring research trends in frailty and delirium in older adults. While Bellelli et al. [8] laid the conceptual groundwork on the frailty–delirium interplay from a clinical-mechanistic perspective, the present study offers a quantitative mapping of research trends, key contributors (countries, institutions, authors, journals), and thematic evolution over time in this same area—providing a complementary rather than redundant level of analysis. Therefore, this study aims to map global publication patterns on frailty and delirium in older adults using bibliometric methods. By revealing descriptive publication patterns and thematic evolution in this field, this study will help researchers understand how frailty and delirium research has developed and where research efforts have concentrated over time. These findings are expected to instigate further investigations by flagging underexplored connections between frailty and delirium that warrant targeted empirical studies. The bibliometric outputs will also provide readers and researchers with a clear intellectual structure, key collaborators, and emerging topics to accelerate research planning and resource allocation.

## 2. Material and Methods

### 2.1. Search Strategies and Data Collection

A literature search on frailty and delirium in older adults was conducted in the Web of Science Core Collection (WoSCC) using a transparent and reproducible bibliometric procedure. WoSCC was selected as the sole data source because it is widely used in bibliometric studies and provides comprehensive citation data together with standardized author, institution, and source information, which supports co-occurrence, collaboration, and citation network analyses [12]. Nevertheless, relying exclusively on the WoSCC may introduce coverage bias due to its over-representation of English-language journals and Western publishers. In bibliometric research, the study corpus is defined by the database and search strategy rather than by individual reassessment of each article against a newly imposed clinical eligibility framework. Accordingly, the present study identified publications through a topic-based search designed to capture the research landscape of frailty and delirium in older adults as represented in the indexed literature. The following search formula was used: ((TS = (Frail* OR weakness OR fatigue OR feebleness OR debility)) AND TS = (Delirium)) AND TS = (“older people” OR “older adult*” OR “older person*” OR “the aged” OR “the elderly” OR “senior*” OR “elder*” OR old-age OR senile OR geriatric) [13,14]. Frailty-related terms were intentionally defined broadly to maximize retrieval sensitivity. This approach was adopted because the literature uses heterogeneous terminology to describe frailty-related vulnerability in older populations, and some relevant studies may not use the single term “frailty” in titles, abstracts, or keywords. The aim of this study was to map the research landscape of frailty and delirium in older adults rather than to restrict the dataset to studies applying one formal frailty model only. However, terms such as “weakness,” “fatigue,” “feebleness,” and “debility” are not strictly synonymous with frailty as a geriatric syndrome and may have reduced search specificity.

No uniform age cutoff was imposed during study selection beyond the terms embedded in the search strategy. This decision reflects the nature of bibliometric analysis, which maps the published literature as indexed, and also acknowledges that definitions of older adults vary across studies and clinical contexts. Some publications define older populations as aged 60 years and above, whereas others use 65 years and above or setting-specific criteria.

Only original articles published in English were included. Reviews, conference abstracts, editorials, letters, case reports, and book chapters were excluded to improve document-type consistency. The search was completed on 11 May 2026 and covered publications from January 2006 to May 2026, with 2026 representing a partial year. The final dataset of 911 publications was obtained through the following steps: (1) literature retrieval in WoSCC using the predefined search strategy, (2) removal of duplicate records, and (3) application of language and document type restrictions. No formal quality assessment or evidence synthesis protocol was applied because the purpose of this study was descriptive bibliometric mapping of publication trends, collaboration patterns, and thematic evolution [9].

### 2.2. Statistical Analysis

Bibliometric visualization and analysis were performed using VOSviewer (version 1.6.20), CiteSpace (version 6.3.R1), and the R package bibliometrix (version 4.4.1). VOSviewer was used to construct and visualize co-occurrence and collaboration networks involving authors, institutions, countries, journals, and keywords. These analyses were used to identify major contributors, collaboration patterns, and thematic structures within the field.

CiteSpace was used to detect keywords with citation bursts, which were interpreted as indicators of emerging or rapidly increasing research attention. Time slicing was set from January 2006 to May 2026, with keywords selected as the node type. A minimum node threshold of 5 was applied for each time slice, and the network was pruned using the Pathfinder and Clip Merge algorithms to improve interpretability. For keyword co-occurrence analysis, a minimum occurrence threshold of 10 was applied to Keywords Plus terms in order to reduce noise from infrequently occurring and weakly connected terms while retaining adequate thematic coverage. Under this threshold, 93 Keywords Plus terms were included in the final network. Cluster structures and overlay visualization were then used to examine thematic groupings and temporal shifts in research focus.

The R package bibliometrix was used to analyze annual publication output, country and institutional productivity, journal performance, and author impact. Research impact was assessed using the h-index, together with the g-index and m-index, to describe productivity and citation performance [15,16]. Journal prestige was evaluated using 2024 Journal Citation Reports (JCR) quartiles and Impact Factor (IF) values. JCR quartiles classify journals into four levels, with Q1 representing the highest citation impact within a subject category. These indicators were used descriptively to characterize the academic influence of journals represented in the dataset. In the interpretation of keyword results, recent burst terms were described as emerging keywords or keywords with recent citation bursts.

## 3. Results

### 3.1. An Overview of Publications

This bibliometric analysis encompassed 911 publications, all of which were research articles indexed in the WoSCC (Figure 1). These articles appeared in 337 different journals from January 2006 to May 2026. The annual growth rate of publications in this field reached 11.18% (Figure 2A).

The annual output of publications on frailty and delirium in older adults demonstrated a clear upward trajectory over the past two decades (Figure 2B). Between 2020 and 2025, the number of articles published each year consistently exceeded 65, reflecting heightened research activity and growing academic interest in this area.

### 3.2. Analysis of the Countries

As shown in Figure 3A and Table 1, the USA dominated the research output on frailty and delirium in older adults, contributing the highest number of articles (185), accounting for 20.3% of the total articles. The USA also led in total publications (TP = 883) and total citations (TC = 8507), with an average citation rate of 46 per article. In this study, “Article” was defined as the number of papers attributed to the country of the corresponding author, whereas “TP” represented the total number of publications counted by country contribution, allowing a single paper to be counted more than once when multiple authors or affiliations were involved.

The collaboration network map shown in Figure 3B illustrates international research partnerships among countries studying frailty and delirium in older adults. Among 32 countries involved in international collaborations with a minimum of 5 articles, the UK demonstrated the strongest global collaboration, with a total link strength of 141.

### 3.3. Analysis of the Institutions

The leading ten institutions in terms of publication volume on frailty and delirium in older adults were displayed in Figure 3C. Harvard University produced the most publications, with a total of 139. Harvard University Medical Affiliates ranked second, contributing 121 publications.

Among the 86 institutions involved in international collaborations with at least 6 published articles, the collaborative network in research on frailty and delirium in older adults was illustrated in Figure 3D. The University of Milano-Bicocca demonstrated the strongest international linkage, with a total link strength of 60. University College London followed closely, exhibiting a total link strength of 59. University of Melbourne also showed significant global cooperation with a total link strength of 53.

### 3.4. Analysis of Journals

The top 20 high-impact journals ranked by h-index were summarized in Table 2. *Age and Ageing* emerged as the leading journal with the highest h-index of 21 (first), publishing 40 publications (second in volume) and accumulating 1301 citations (second in TC), alongside an IF of 6.0 (Q1). *BMC Geriatrics* ranked first in publication count with 52 articles, held an h-index of 16, and received 567 citations, with an IF of 3.4 (Q2).

The co-occurrence network of journals included 75 journals, each with at least 3 occurrences (Figure 4A). Among these, *Age and Ageing* demonstrated the strongest total link strength at 122. It was followed by *Anesthesia and Analgesia* with a total link strength of 77, and *BMC Geriatrics* with 59. Similarly, the coupling network of journals also comprised 75 journals, each having a minimum of two coupling links (Figure 4B). In this network, *BMC Geriatrics* exhibited the highest total link strength of 11,528. *Age and Ageing* ranked second with a total link strength of 6404, followed by *BMJ Open* with 5044.

### 3.5. Analysis of the Authors

The top 20 authors in this research area were ranked by their h-index, as detailed in Table 3. Bellelli Giuseppe led with the highest number of publications (20, ranked first) and an h-index of 8, accumulating 229 citations. Inouye Sharon K. followed with 9 articles, the highest h-index of 8, and 365 citations.

The collaboration network among authors engaged in international partnerships with at least 5 publications was depicted in Figure 4C. Among the 59 authors meeting this criterion, Bellelli, Giuseppe exhibited the highest total link strength of 53. Morandi, Alessandro ranked second with a total link strength of 41, followed by Ferrara, Maria Cristina with 26.

### 3.6. Analysis of the Keywords

In total, 93 keywords with a minimum of 10 occurrences were identified in keyword co-occurrence analysis. This study identified six distinct clusters based on the analysis of 93 keywords (Figure 5A). The red cluster, comprising 27 items, primarily addressed adverse outcomes and comprehensive geriatric assessment, with keywords such as “index,” “care,” and “depression.” The green cluster, containing 24 items, centered on cognitive decline and neuropsychiatric disorders. High-frequency terms included “cognitive decline,” “anesthesia,” and “functional decline.” The blue cluster, with 17 items, emphasized perioperative and intensive care settings, featuring keywords such as “critical illness,” “ICU,” and “cardiac surgery.” Yellow cluster, consisting of 10 items, focused on postoperative delirium and geriatric complications, including “hip fracture,” “falls,” “disability,” and “risk factors.” The purple cluster, containing 8 items, was oriented toward prediction and validation, with terms such as “prediction,” “validation,” and “tool.” The cyan cluster, comprising 7 items, addressed epidemiology and management-related themes, including “guidelines,” “prevalence,” and “management.”

The network visualization of keyword co-occurrence was presented in Figure 5B. In the latest period (after 2021, yellow), novel themes appeared, exemplified by terms like “surgery,” “complications,” and “mortality,” as well as “enhanced recovery” and “preoperative frailty.” This shift indicated a growing focus on perioperative care, optimization of surgical outcomes, and the role of frailty assessment in clinical decision-making.

### 3.7. Analysis of Burst Keywords

The evolution of research trends in frailty and delirium in older adults from January 2006 to May 2026 was marked by the sequential emergence of burst keywords (Figure 5C). In the initial phase (2006–2014), terms such as “care” (strength = 2.68, 2006–2007), “intervention” (10.37, 2006–2015), “confusion assessment method” (7.39, 2009–2016), and “disability” (3.52, 2009–2014) dominated, reflecting an early emphasis on clinical assessment tools, intervention strategies, and functional decline. Between 2015 and 2020, keywords like “colorectal surgery” (3.20, 2015–2019), “people” (4.81, 2017–2019), “prevalence” (3.51, 2015–2017), and “functional decline” (6.26, 2011–2016) gained prominence, indicating a growing focus on surgical outcomes, epidemiological patterns, and healthcare resource utilization. From 2018 onward, the field shifted toward patient-centered and methodological themes, with “quality of life” (3.44, 2019–2021), “meta-analysis” (3.45, 2020–2022), “morbidity” (3.10, 2020–2022), and “age” (3.53, 2021–2022) reflecting an increased attention to comprehensive evaluation and evidence synthesis. Most recently, from 2021 to 2026, emerging keywords such as “health” (3.11, 2021–2023), “reliability” (2.93, 2021–2022), “validity” (2.75, 2021–2022), “impairment” (2.65, 2022–2023), “cardiac surgery” (4.02, 2023–2024), “scale” (3.96, 2024–2026), “anesthesia” (3.19, 2022–2026), and “American college” (2.64, 2024–2026) highlighted a shift toward age-related health issues, perioperative management, and specialized surgical care for older adults.

## 4. Discussion

### 4.1. General Information

As a bibliometric investigation, this study revealed sustained global scholarly engagement in frailty and delirium research, characterized by exponential growth and thematic diversification over two decades. Annual publication outputs demonstrated a steady rise from 6 articles in 2006 to a peak of 118 in 2025. The analysis encompassed 911 research articles involving 6805 authors from 337 journals, illustrating the multidisciplinary nature of this field and its integration across geriatrics, neurology, psychiatry, and surgical specialties. Geographically, the USA dominated research productivity, contributing 20.3% of total publications and achieving the highest citation impact. Institutional contributions were spearheaded by Harvard University. The journal *Age and Ageing* emerged as the most influential outlet.

### 4.2. Red Cluster: Adverse Outcomes and Comprehensive Geriatric Assessment

The red cluster primarily addressed adverse outcomes and comprehensive geriatric assessment. The co-occurrence of these terms reflects a conceptual linkage in which geriatric status—measured through various frailty indices—is increasingly viewed as a predictor of downstream outcomes, while “care” and “depression” situate this relationship within broader patient-centered and mental health contexts. Temporally, terms within this cluster appeared steadily throughout the study period without a distinct burst, which, from a bibliometric perspective, suggests that comprehensive geriatric assessment has been a consistent foundational theme rather than an emerging one. This pattern indicates that the field has long recognized the importance of quantifying geriatric vulnerability, but only in more recent years has this quantification appeared more frequently in studies linked to delirium outcomes. The bibliometric pattern itself does not establish the clinical utility of these assessments; however, when contextualized with clinical evidence, it helps identify areas where empirical research has concentrated. For example, clinical studies have reported that a higher frailty index is associated with increased incidence and longer duration of delirium in hospitalized older adults [17]. Similarly, Song et al. found that frailty-related impairment in activities of daily living mediates depressive symptoms, suggesting that functional assessment is integral to understanding the full clinical picture of frail older adults [18].

### 4.3. Green Cluster: Cognitive Decline and Neuropsychiatric Disorders

The green cluster centered on cognitive decline and neuropsychiatric disorders. The co-occurrence of cognitive and functional decline with anesthesia indicates that this cluster captures a peri-exposure conceptual framework—cognitive and functional trajectories are examined in relation to external stressors such as anesthetic exposure. Notably, “anesthesia” emerged as a burst keyword in the most recent period (2022–2026), suggesting that scholarly attention may have shifted toward the intersection of anesthetic management and cognitive outcomes. From a bibliometric perspective, this temporal pattern suggests a growing research focus on modifiable perioperative factors that may be associated with cognitive outcomes in frail older adults [19]. This interpretation is consistent with, but should not be conflated with, clinical evidence: meta-analyses have reported that preoperative physical frailty is associated with an increased risk of postoperative delirium, which underscores the clinical relevance of frailty screening in perioperative settings [20]. However, the cluster also suggests that functional decline may not be fully captured by frailty alone, as prospective evidence indicates that prediction of functional decline may require additional factors beyond baseline frailty [21].

### 4.4. Blue Cluster: Perioperative and Intensive Care Settings

The blue cluster emphasized perioperative and intensive care settings. The strong co-occurrence of these terms bibliometrically reflects that frailty and delirium are most intensively studied in high-acuity environments, where physiological stress, procedural burden, and care intensity converge as research contexts. “Cardiac surgery” appeared as a recent burst keyword (2023–2024), suggesting that cardiovascular surgical populations have emerged as a particularly active investigative context within this broader cluster. This bibliometric pattern may reflect growing research attention to the prevalence of frailty in older cardiac surgery patients and the occurrence of delirium as a complication in this setting [22]. Importantly, however, the bibliometric pattern itself does not imply a causal relationship between cardiac surgery and delirium; it merely indicates that this clinical context has attracted substantial research interest. Observational clinical studies have identified frailty index, lower preoperative MMSE scores, and longer operation time as factors associated with postoperative delirium in frail elderly patients undergoing on-pump cardiac surgery [23]. The cluster also underscores the need for precision-based approaches to delirium prevention in ICUs, where frailty, advanced age, sedation, and sepsis are key risk factors [24].

### 4.5. Yellow Cluster: Postoperative Delirium and Geriatric Complications

The yellow cluster focused on postoperative delirium and geriatric complications. This tight cluster indicates that frailty–delirium research is strongly anchored to clinically meaningful postoperative events, with hip fracture serving as a paradigmatic clinical model. Unlike other clusters, this group of keywords has been consistently present throughout the study period without strong temporal bursts, suggesting that postoperative delirium in orthopedic populations represents a mature and well-established line of inquiry rather than an emerging one. The persistent co-occurrence of “disability” and “risk factors” with “hip fracture” implies that the field has moved beyond identifying simple associations toward characterizing the downstream functional consequences of delirium in this vulnerable population [25]. Cross-sectional evidence supports this pattern: higher frailty in older hip fracture patients is associated with functional dependence, postoperative delirium, and adverse outcomes including ICU admission and mortality [26]. Furthermore, frailty risk independently predicts substantially worse outcomes—up to ~6-fold higher in-hospital mortality odds and prolonged length of stay—in older adults undergoing hip fracture surgery [27].

### 4.6. Purple Cluster: Prediction and Validation Tools

The purple cluster was oriented toward prediction and validation. The emergence of this cluster as a distinct thematic group—relatively small but clearly defined—reflects a methodological shift toward operationalizing frailty-related constructs into measurable instruments aimed at forecasting delirium. Notably, “scale” and “validity” appeared as recent burst keywords (2024–2026), indicating that tool development and psychometric evaluation are currently active frontiers within the field. This temporal trend suggests that the research community is moving beyond establishing associations between frailty and delirium toward developing clinically deployable risk stratification instruments [28]. For instance, inclusion of frailty significantly improved the accuracy of delirium prediction models in cardiac intensive care units [29], and interpretable machine learning models have been constructed for early delirium prediction in older ICU patients, with interpretability enabling bedside decision support [30]. The validation emphasis within this cluster supports the translation of research findings into actionable clinical tools.

### 4.7. Cyan Cluster: Epidemiology and Management-Related Themes

The cyan cluster addressed epidemiology and management-related themes. This cluster, though the smallest, captures an important translational dimension: beyond understanding mechanisms and risk factors, the field is increasingly focused on quantifying disease burden and translating evidence into actionable clinical approaches. “Prevalence” appeared as an early burst keyword (2015–2017), whereas “guidelines” and “management” have shown more sustained presence, suggesting that epidemiological characterization preceded the development of structured clinical guidance. This chronological pattern implies that the field has matured from descriptive prevalence studies toward implementation-oriented research. Meta-analytic evidence estimated frailty prevalence at 34% and delirium at 21% in hospitalized older adults, and found that frailty independently increases delirium risk (RR ≈ 1.66), providing quantitative evidence that frailty is a potential management target for prevention strategies [1]. The stable prevalence of delirium over four decades, as documented by Gibb et al. [31], further emphasizes that delirium should be treated as an enduring management priority in elderly care pathways, with stronger attention to consistent guideline-based diagnosis and improved detection strategies.

In addition to the six thematic clusters, the keyword burst and clustering results revealed three additional layers of insight regarding the field’s evolution.

For newly evolved keywords, terms such as “anesthesia” (burst: 2022–2026), “cardiac surgery” (burst: 2023–2024), and “scale” (burst: 2024–2026) suggest growing interest in perioperative anesthetic management, specialized surgical populations, and standardized risk assessment approaches. This pattern is consistent with contemporary perioperative brain-health and delirium prevention frameworks, and signals that the field is increasingly focusing on modifiable intraoperative and postoperative factors rather than merely describing risk [32].

For the low-frequency but clinically relevant keywords, those that may be clinically important yet overshadowed by the broader frailty construct, our analysis highlighted sarcopenia, gait speed, and malnutrition. These terms did not appear with sufficient frequency to form independent clusters, yet they point to more proximal, measurable, and potentially modifiable physiological drivers underlying frailty-related vulnerability. Importantly, existing evidence supports that sarcopenia is associated with postoperative delirium, preoperative malnutrition independently predicts delirium, and slow gait speed is linked to increased delirium risk [33,34]. The finding suggests that future research should consider disaggregating frailty into its component phenotypes to identify more targeted prevention strategies.

Finally, keywords with low co-occurrence identified in the analysis, such as pain, depression, colorectal cancer, and replacement (e.g., arthroplasty/major orthopedic replacement), indicate potential gaps in the literature. These terms are notably absent from the dominant frailty–delirium research narrative, suggesting that symptom-specific (psychological factors and pain burden) and procedure-specific contexts are comparatively underintegrated. Given that preoperative pain and depressive symptoms have been shown to be associated with postoperative delirium, and that delirium incidence varies substantially across surgical procedures, including colorectal and orthopedic surgeries [35], our bibliometric findings highlight opportunities for future studies to more explicitly incorporate these domains into risk prediction models and prevention strategies to better reflect mechanistic heterogeneity and improve translational relevance.

### 4.8. Limitations

This study had several limitations. First, the analysis was restricted to publications indexed in the Web of Science Core Collection, which may have excluded relevant studies from other databases. Because WoSCC tends to provide broader coverage of journals and research from developed countries, the resulting dataset may more strongly reflect research activity in developed settings. The findings should therefore be interpreted cautiously when considering global representativeness. Second, only English-language publications were included, which may have introduced language bias and reduced the visibility of work published in other languages. Third, because bibliographic databases are continuously updated, some recently published studies may not have been captured at the time of retrieval. Fourth, the search strategy used broad frailty-related terms, including “weakness,” “fatigue,” “feebleness,” and “debility,” in order to maximize retrieval sensitivity. This choice was made because frailty-related vulnerability is described inconsistently across the literature. However, these terms are not fully equivalent to frailty as a geriatric syndrome and may have introduced some nonspecific records. As a result, the dataset should be understood as representing the indexed research landscape around frailty-related vulnerability and delirium in older adults rather than a corpus restricted only to studies using one standardized frailty definition. Fifth, no uniform age cutoff such as 60 years or 65 years was imposed during study selection beyond the search terms themselves. This reflects the bibliometric design of the study, in which the literature is identified through indexed terminology rather than reclassified according to a single externally applied clinical definition. Nevertheless, definitions of older adults vary across studies, and this heterogeneity may have affected the comparability of the included literature. Sixth, the explanation of keyword analysis may involve a degree of subjectivity. Although clustering was generated algorithmically, the interpretation and naming of clusters depended on the researchers’ judgment. In addition, the results are sensitive to parameter settings, including the minimum keyword occurrence threshold of 10, the time-slice node threshold of 5, and the use of Pathfinder and Clip Merge pruning in CiteSpace. Different parameter choices might have yielded somewhat different thematic structures. Seventh, bibliometric indicators such as citation counts, burst strength, and the h-index reflect scholarly attention rather than methodological rigor or clinical importance. Highly cited articles are not necessarily the most robust or practice-changing, and recent high-quality studies may appear less influential because they have had less time to accumulate citations. Eighth, name disambiguation problems in author and institutional data may have affected the accuracy of collaboration analyses. Finally, this study included only original articles and excluded document types such as reviews, conference abstracts, preprints, and dissertations. Consequently, secondary evidence and emerging findings not yet represented in indexed original research may have been omitted from this bibliometric mapping.

## 5. Conclusions

This bibliometric analysis maps the dynamic research landscape surrounding frailty and delirium in older adults, identifying six distinct thematic clusters: adverse outcomes and comprehensive geriatric assessment, cognitive decline and neuropsychiatric disorders, perioperative and intensive care settings, postoperative delirium and geriatric complications, prediction and validation tools, and epidemiology with management-oriented guidance. Moreover, the temporal trends highlight emerging priorities in perioperative care, emphasizing the need for tailored interventions that address frailty and delirium to enhance recovery and improve outcomes.

## Figures and Tables

**Figure 1:**
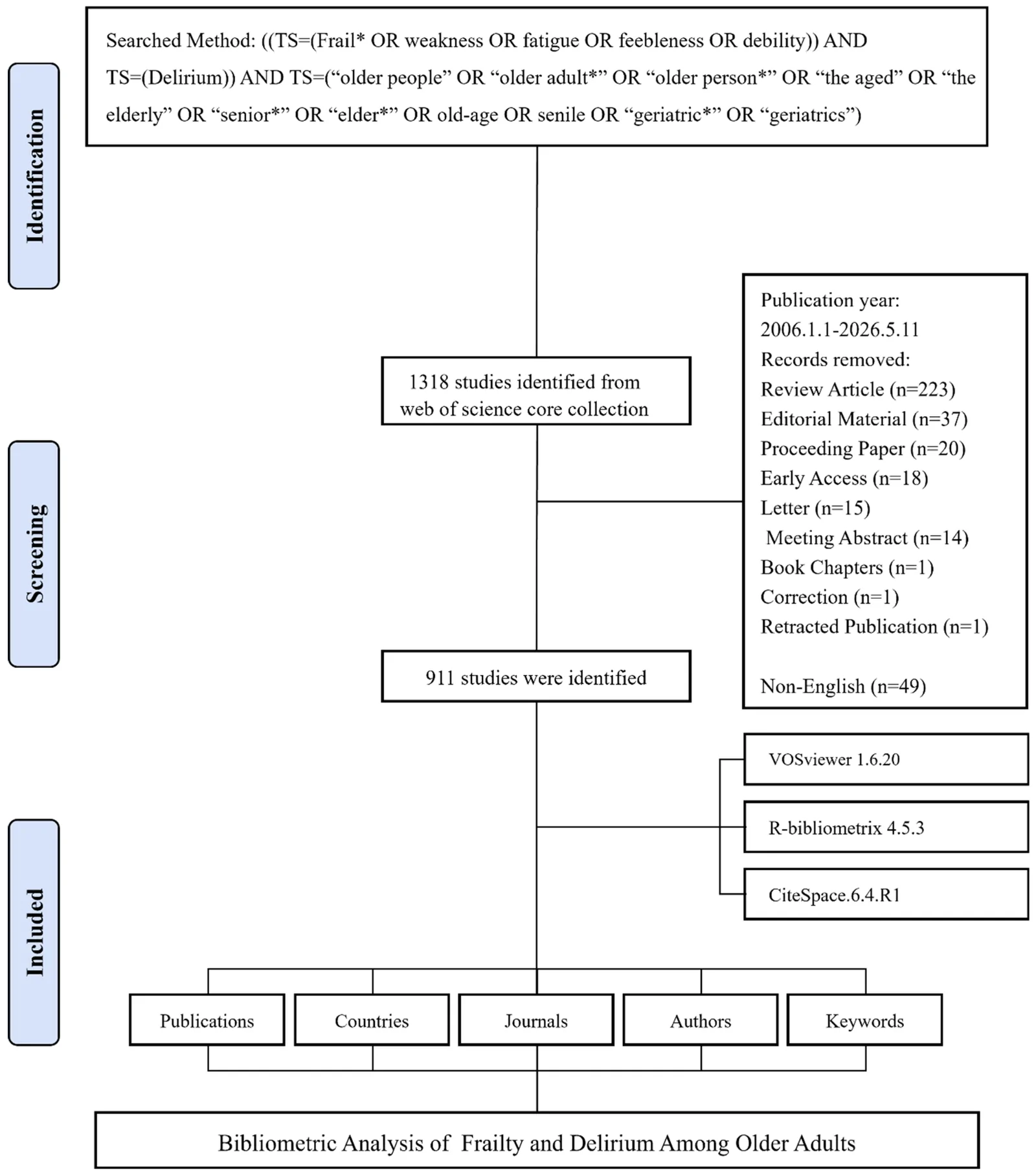
Flowchart of the literature screening process for frailty and delirium in older adults.

**Figure 2:**
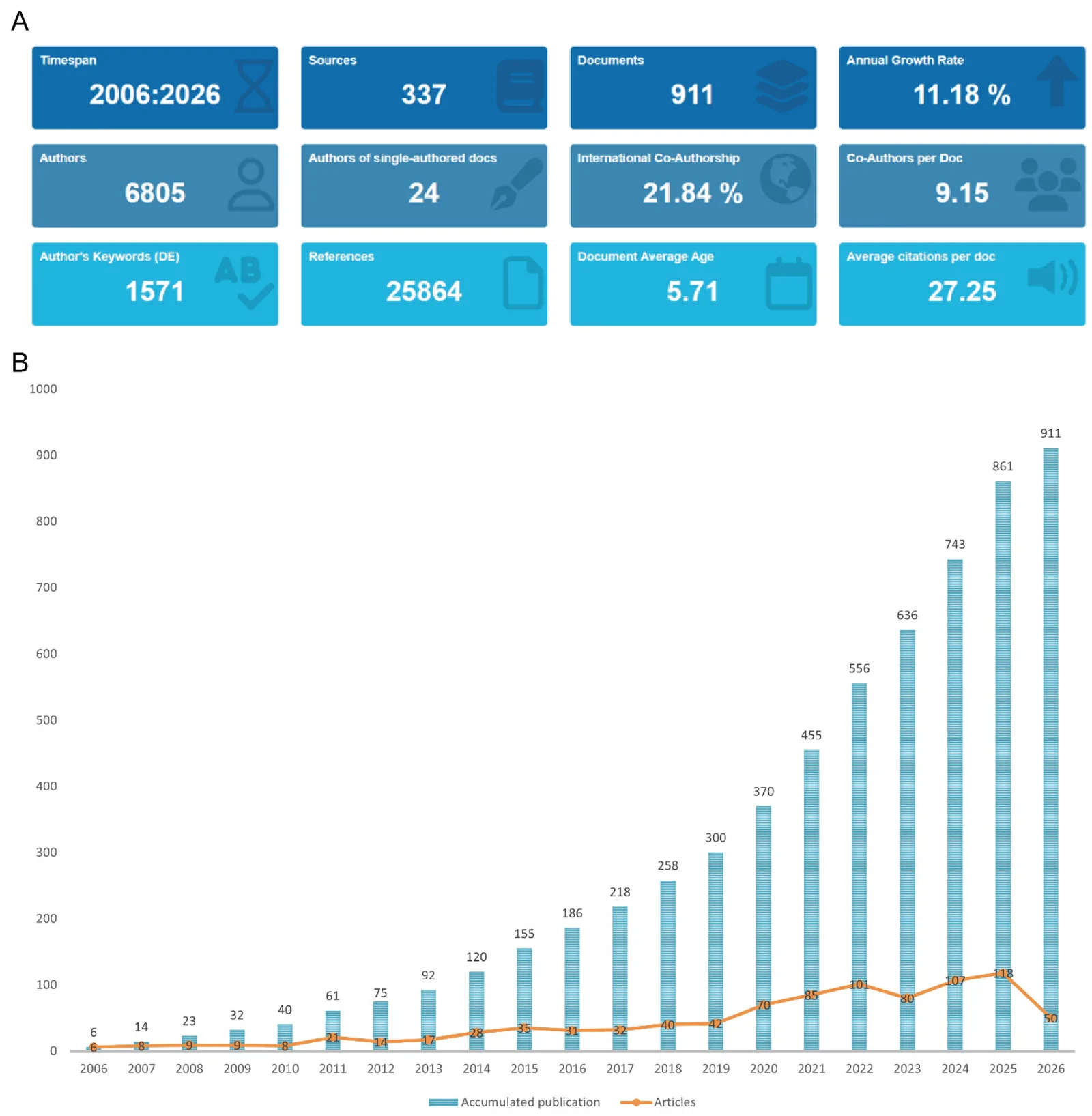
Overall research landscape and annual publication trends in frailty and delirium in older adults. (**A**) General overview of the research field of frailty and delirium in older adults. (**B**) Annual number of publications on frailty and delirium in older adults.

**Figure 3:**
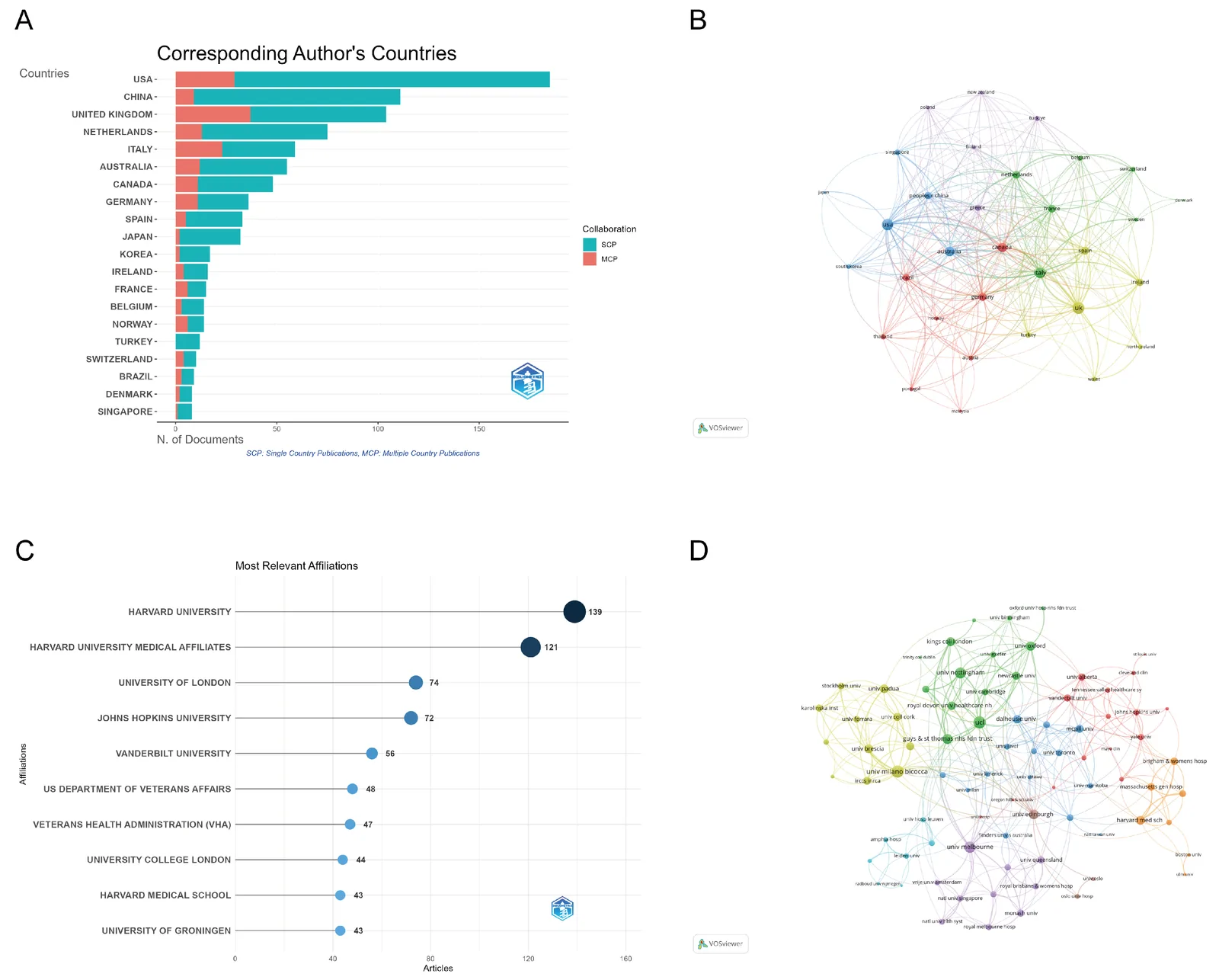
Analysis of countries and institutions. (**A**) Distribution of corresponding author’s publications by country. SCP: Single Country Publications. MCP: Multiple Country Publications. (**B**) Visualization map depicting the collaboration among different countries. (**C**) Top ten institutions by article count and rank. Circle size shows article count. Darker shades indicate higher ranks. (**D**) Visualization map depicting the collaboration among different institutions.

**Figure 4:**
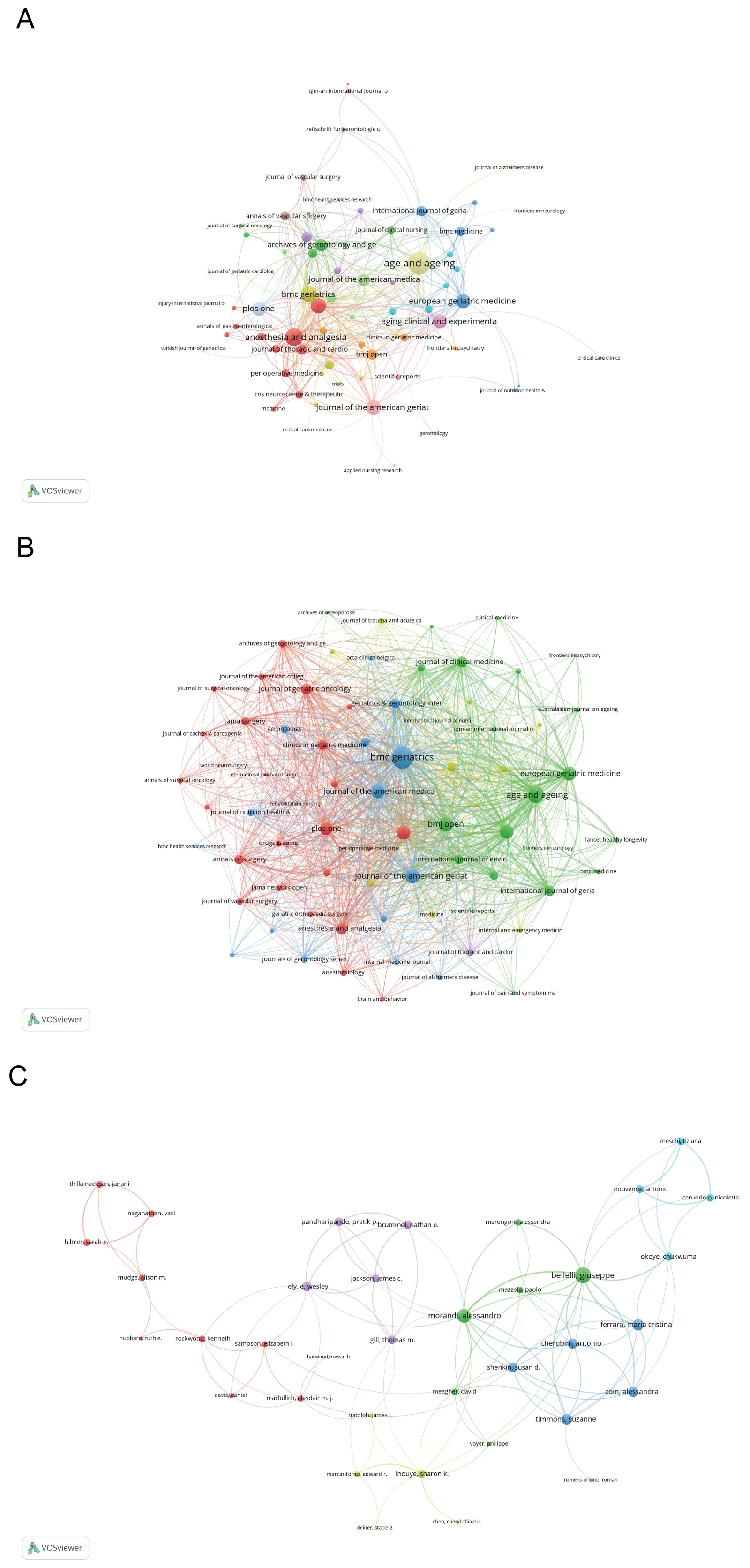
Analysis of journals. (**A**) Journal co-occurrence network diagram. (**B**) Journal bibliography coupling network diagram. (**C**) Visualization map depicting the collaboration among different authors.

**Figure 5:**
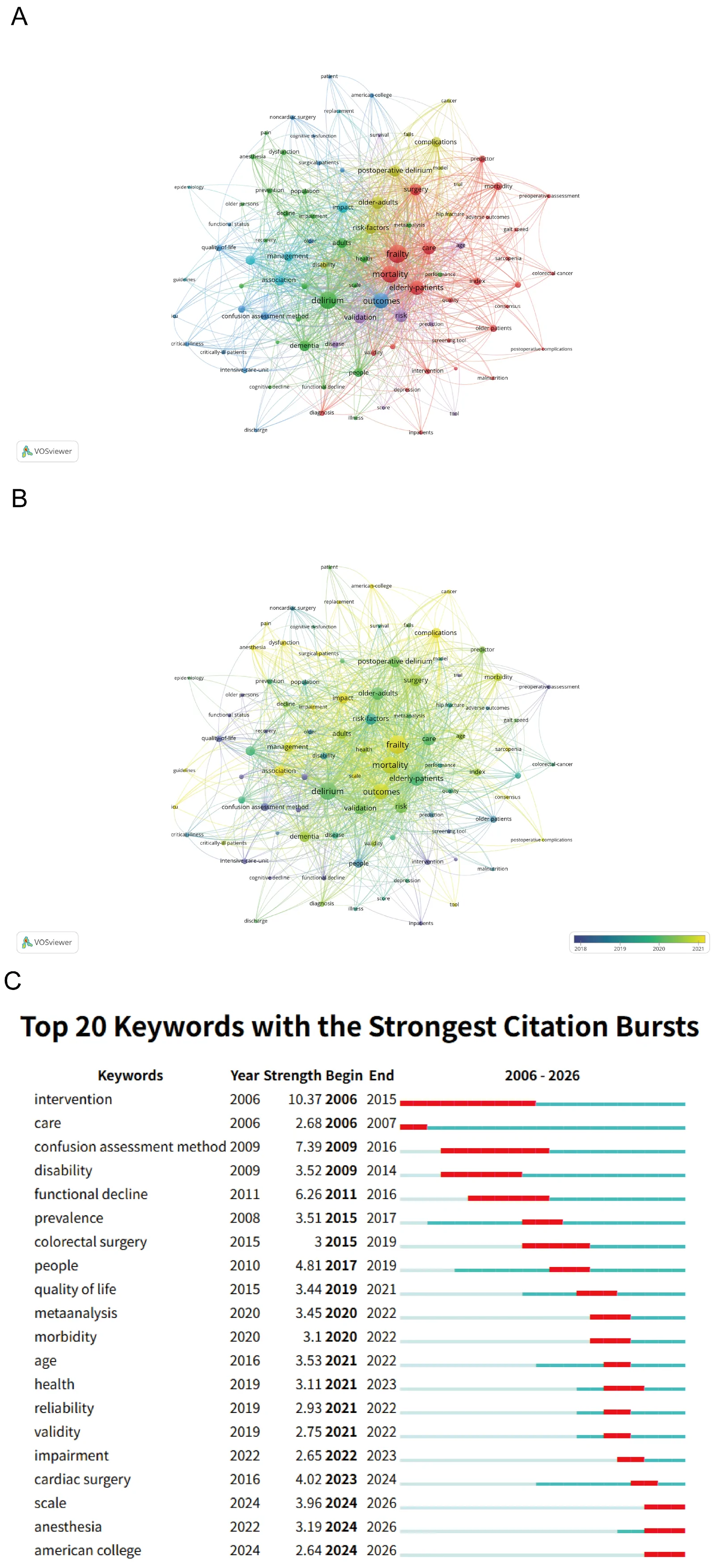
Visual analysis of keywords. (**A**) Keyword cluster analysis. (**B**) Visual analysis of keyword co-occurrence network analysis. (**C**) Top 20 keywords with the strongest citation bursts.

**Table 1:** Publication and citation profiles of leading countries.

Country	Articles	Freq	SCP	MCP	MCP_Ratio	TP	TP_Rank	TC	TC_Rank	Average Citations
USA	185	0.203	156	29	0.157	883	1	8507	1	46
CHINA	111	0.122	102	9	0.081	384	4	1141	7	10.3
UNITED KINGDOM	104	0.114	67	37	0.356	728	2	3631	2	34.9
NETHERLANDS	75	0.082	62	13	0.173	344	5	2025	3	27
ITALY	59	0.065	36	23	0.390	407	3	1184	6	20.1
AUSTRALIA	55	0.060	43	12	0.218	335	6	1661	4	30.2
CANADA	48	0.053	37	11	0.229	290	7	1502	5	31.3
GERMANY	36	0.040	25	11	0.306	218	8	558	10	15.5
SPAIN	33	0.036	28	5	0.152	213	9	633	9	19.2
JAPAN	32	0.035	30	2	0.063	134	10	410	13	12.8
SOUTH KOREA	17	0.019	15	2	0.118	80	14	639	8	37.6
IRELAND	16	0.018	12	4	0.250	106	13	322	14	20.1
FRANCE	15	0.016	9	6	0.400	126	11	437	12	29.1
BELGIUM	14	0.015	11	3	0.214	79	15	456	11	32.6
NORWAY	14	0.015	8	6	0.429	62	16	308	15	22
TURKEY	12	0.013	12	0	0.000	32	22	75	25	6.2
SWITZERLAND	10	0.011	6	4	0.400	44	17	104	22	10.4
BRAZIL	9	0.010	6	3	0.333	112	12	130	20	14.4
DENMARK	8	0.009	6	2	0.250	37	19	160	16	20
SINGAPORE	8	0.009	7	1	0.125	36	20	115	21	14.4

Articles: Publications of Corresponding Authors only. Freq: Frequency of Total Publications. MCP_Ratio: Proportion of Multiple Country Publications. TP: Total Publications. TP_rank: Rank of Total Publications. TC: Total Citations. TC_rank: Rank of Total Citations. Average Citations: The average number of citations per publication. “Article” was defined as the number of papers attributed to the country of the corresponding author, whereas “TP” represented the total number of publications counted by country contribution, allowing a single paper to be counted more than once when multiple authors or affiliations were involved.

**Table 2:** Bibliometric indicators of high-impact journals.

Journal	H-Index	G-Index	M-Index	IF 2023	JCR 2023	TP	TP Rank	TC	TC Rank	First Publication Year
***Age and Ageing***	21	40	1.000	6.0	Q1	40	2	1301	2	2006
***BMC Geriatrics***	16	28	0.941	3.4	Q2	52	1	567	7	2010
***Journal of the American Medical Directors Association***	14	23	0.667	4.2	Q2	23	6	745	5	2006
***Journal of the American Geriatrics Society***	12	23	0.600	4.3	Q1	23	5	2205	1	2007
***Anesthesia & Analgesia***	10	11	0.625	4.6	Q1	11	13	426	9	2011
***Clinical Interventions in Aging***	10	19	0.769	3.5	Q2	21	8	251	25	2014
***Aging Clinical and Experimental Research***	10	17	0.526	3.4	Q2	22	7	241	27	2008
***European Geriatric Medicine***	10	17	0.714	3.5	Q2	26	3	152	34	2013
***PLOS ONE***	9	15	0.643	2.9	Q1	15	10	343	15	2013
***International Journal of Geriatric Psychiatry***	8	9	0.571	3.6	Q1	9	17	260	23	2013
***BMJ Open***	7	15	0.636	2.4	Q1	24	4	252	24	2016
***Journal of Geriatric Oncology***	7	16	0.467	3.0	Q2	16	9	143	37	2012
***Geriatrics & Gerontology International***	7	12	0.500	2.4	Q2	12	12	134	42	2013
***European Journal of Surgical Oncology (EJSO)***	7	9	0.583	3.5	Q1	9	16	70	86	2015
***Trials***	7	11	0.538	2.0	Q3	11	14	24	248	2014
***Clinics in Geriatric Medicine***	6	10	0.353	3.1	Q3	10	15	156	33	2010
***Journal of Clinical Nursing***	6	6	0.545	3.2	Q1	6	30	107	62	2016
***Gerontology***	6	8	0.286	3.1	Q3	8	21	60	102	2006
***Frontiers in Aging Neuroscience***	6	8	1.000	4.1	Q2	8	19	58	107	2021
***Annals of Surgery***	5	6	0.417	7.5	Q1	6	25	395	12	2015

Notes: H_index: The h-index of the journal, which measures both the productivity and citation impact of the publications. g_index: The g-index of the journal, which gives more weight to highly-cited articles. m_index: The m-index of the journal, which is the h-index divided by the number of years since the first published paper. TP: Total Publications. TP_rank: Rank of Total Publications. TC: Total Citations. TC_rank: Rank of Total Citations. Average Citations: The average number of citations per publication. PY_start: Publication Year Start, indicating the year the journal started publication.

**Table 3:** Publication and citation profiles of high-impact authors.

Authors	h_Index	g-Index	m-Index	PY_Start	TP	TP_Frac	TP_Rank	TC	TC_Rank
BELLELLI GIUSEPPE	8	15	0.67	2015	20	1.89	1	229	27
INOUYE SHARON K.	8	9	0.38	2006	9	1.29	4	365	17
ROCKWOOD KENNETH	8	10	0.38	2006	10	1.55	3	539	6
DAVIS DANIEL	7	8	0.78	2018	8	0.54	6	337	19
ELY E. WESLEY	6	8	0.33	2009	8	0.82	7	685	5
HUBBARD RUTH E.	6	7	0.35	2010	7	0.52	15	521	7
JACKSON JAMES C.	6	6	0.33	2009	6	0.26	25	1058	1
KIM CHEOL-HO	6	7	0.43	2013	7	1.19	16	513	8
KIM KWANG-IL	6	7	0.43	2013	7	1.76	17	513	8
MORANDI ALESSANDRO	6	12	0.33	2009	12	1.07	2	425	12
SAMPSON ELIZABETH L.	6	7	0.43	2013	7	0.82	20	217	30
VAN DER LAAN LIJCKLE	6	9	0.50	2015	9	1.26	5	373	16
VAN LEEUWEN BARBARA L.	6	6	0.40	2012	6	0.88	32	381	15
WELCH CARLY	6	7	0.75	2019	7	0.64	22	404	13
DEINER STACIE G.	5	7	0.71	2020	7	0.82	12	207	32
EGUCHI HIDETOSHI	5	7	0.56	2018	7	0.98	13	120	40
GILL THOMAS M.	5	8	0.31	2011	8	1.09	8	717	4
MACLULLICH ALASDAIR M. J.	5	6	0.38	2014	6	0.84	9	395	14
MARCANTONIO EDWARD R.	5	8	0.42	2015	8	0.19	27	72	47
MASOLI JANE	5	7	0.45	2016	7	0.50	47	219	29

Notes: H_index: The h-index, which measures both the productivity and citation impact of the publications. g_index: The g-index, which gives more weight to highly-cited articles. m_index: The m-index, which is the h-index divided by the number of years since the first published paper. TP: Total Publications. “TP_Frac” refers to fractional total publications, in which each publication is counted proportionally across contributing countries, institutions, or authors, so that the total weight of one publication equals 1. TP_rank: Rank of Total Publications. TC: Total Citations. TC_rank: Rank of Total Citations. PY_start: Publication Year Start, indicating the year the author started publishing.

## Data Availability

No new data were created or analyzed in this study. Data sharing is not applicable to this article.
